# A Cure for Small-Pox

**Published:** 1881-11

**Authors:** 


### A CURE FOR SMALLPOX.

I herewith append a recipe, a Paris physician says, which has been used to my knowledge in hundreds of cases. It will prevent or cure the smallpox though the pittings are filling. When Jenner discovered cowpox in England, the world of science hurled an avalanche of fame upon his head, but when the most scientific school of medicine in the world—that of Paris—published the recipe as a panacea for smallpox, it passed unheeded. It is as unfailing as fate, and conquers in every instance. It is harmless when taken by a well person. It will also cure scarlet fever. Here is the recipe as I have used it, and cured my children of scarlet fever; here it is as I have used it to cure the smallpox; when learned physicians said the patient must die, it cured. Sulphate of zinc, one grain; foxglove (digitalis), one grain; half a teaspoonful of sugur; mix with two tablespoonfuls of water. When thoroughly mixed, add four ounces of water. Take a spoonful every hour. Either disease will disappear in twelve hours. For a child, smaller doses, according to age. If counties would compel their physicians to use this there woutd be no need of pest-houses. If you value advice and experience, use this for that terrible disease.

